# Effects of Secondary Porosity on Microstructure and Mechanical Properties of SAP-Containing Lime-Based Plasters

**DOI:** 10.3390/polym14061162

**Published:** 2022-03-15

**Authors:** Jan Fořt, Martin Böhm, Igor Medveď, Martin Mildner, Robert Černý

**Affiliations:** Department of Materials Engineering and Chemistry, Faculty of Civil Engineering, Czech Technical University in Prague, Thákurova 7, Prague 6, 166 29 Prague, Czech Republic; martin.bohm@fsv.cvut.cz (M.B.); igor.medved@fsv.cvut.cz (I.M.); martin.mildner@fsv.cvut.cz (M.M.); cernyr@fsv.cvut.cz (R.Č.)

**Keywords:** superabsorbent polymer, microstructure, secondary porosity, affected zone, lime-based plaster, mechanical strength

## Abstract

Despite the many benefits associated with the utilization of superabsorbent polymers (SAPs), several drawbacks have been reported. In particular, the effect of SAPs on microstructure, together with its consequences for mechanical properties, is not fully understood yet for some composite materials. This study analyzes the role of SAPs in the formation of the microstructure of lime composites, taking into account their chemical composition. The obtained experimental results show that the particle size and cross-linking density of used SAPs are crucial parameters affecting both the microstructure and mechanical performance of the analyzed composites. Coarser SAPs with low cross-linking density in the dosage of 0.5 and 1 wt.% are found as the most suitable solution, leading even to a slight improvement of mechanical parameters. The secondary porosity formed by swelled hydrogels is identified as a very significant factor since hydrogel-filled voids do not contribute to the strength parameters. The formation of the affected zone around SAP cores depends on the chemical composition of SAPs considerably as the higher cross-linking density influences the desorption rate. Based on achieved results, utilization of SAPs in building materials should be studied at a more detailed level with particular importance on the definition of SAP-related voids and affected zone around SAP particles.

## 1. Introduction

The utilization of superabsorbent polymers (SAPs) in the field of building materials engineering represents a promising research line that provides significant improvements in the sense of building materials performance [1,2,3]. Specifically, SAPs are very interesting as a concrete admixture due to their capability to reduce the autogenous shrinkage, improve the freeze–thaw resistance, and promote the self-healing and self-sealing of cracks [4,5]. Apart from the utilization in high-performance concrete types, their potential lies also in moderation of indoor relative humidity levels secured by the large water retaining capacity, in other words, for modification of interior plasters [6,7,8,9] in order to achieve optimal interior conditions in terms of relative humidity. The incorporation of SAPs also has an eminent effect on the material microstructure. The side effects are related to modification of fresh mixture rheology [10], microstructure formation [11], penetration of chloride ions [12], or aggressive environment resistance [13]. The effect of internal curing based on modified water migration due to water retention, release, and reswelling governed by SAP particles represents a crucial phenomenon that needs to be described in detail. In this regard, the pore structure was studied extensively since the cementitious matrix formation substantially depends on the type and amount of used SAPs, as concluded in several research papers [11,14]. Considering the importance of formed voids on the functional properties of building materials, the detailed understanding of the microstructure of material represents a very important issue. Therefore, the influence of the chemical composition of SAPs, cross-linking density, particle size, and pH sensitivity was studied extensively to provide guidelines for such materials’ design [1,15]. The knowledge of phase and pore distribution is necessary for understanding the chemical and physical properties and prediction of a material’s performance or service life. Since the formation of voids in cementitious materials is driven by the swelling of SAPs, the absorption and desorption rate plays an important role in microstructure formation [16]. However, the effect of SAPs on mechanical performance is rather two-fold. While the strength can be improved thanks to the mitigation of shrinkage occurrence and crack formation, the inappropriate dosages of SAPs may result in loss of mechanical strength [3].

Various microstructure-based models were introduced to describe the transport properties, pore size distribution, and mechanical strength of SAP-modified composite materials [17,18]. As it follows from the studies performed, SAPs applied in building materials are responsible for larger void formation due to their swelling. Consequently, water released during material maturing induces further modifications in material microstructure by voids formation and an increase in total porosity. Yang et al. [19] reported that released water may induce hydration of nonhydrated binder particles and more distinct changes can be observed at later ages, compared to the reference material without SAP. However, the issue of the affected zone was studied only rarely since the used experimental techniques may limit the obtained results [20]. For example, Mechtcherine et al. [21] did not find any distinct modifications in the cement paste around SAP particles by using backscattered electron imaging. On the other hand, pronounced changes driven by continuous hydration of unreacted particles during the release of entrained water from SAP cores were accompanied by significant effects on the properties of the SAP surrounding area. 

A majority of research efforts aimed at lime-based composites for ambient moisture moderation have been focused on the description of the effects of SAPs on rheologic properties [9,22], moisture storage, and moisture buffering [8,23,24], but the microstructure formation of lime composites modified by SAPs remained overlooked. Considering the complexity of the transport process in porous building materials, the material characteristics of such modified composites should be subjected to more detailed research. In this regard, Isikdag and Topcu [25] pointed out the particular importance of understanding the interconnection between microstructural characteristics and functional properties of lime mortars, including the moisture transport and storage properties.

In understanding the role of SAPs in lime-based plasters, their effect on microstructure formation, in particular, is a principal factor for effective material design. The utilization of appropriate techniques represents the rule of the thumb in that respect. Major gaps in the current literature can be found in limited attention paid to the role of voids formed by SAP swelling, and consequences in mechanical parameters. Moreover, understanding the SAP-filled voids as dense material may lead to several misrepresentations of material performance, especially in the case of low-porosity composites. Taking into account the current gap in the available knowledge, this study contemplates the formation of the microstructure of lime-based plasters modified by three types of SAPs. The chemical composition and particle size distribution of selected SAPs are considered, in order to provide advanced knowledge on the incorporation of SAPs into the lime matrix. Particular attention is paid to the formation of porous space, as affected by the chemical characteristics of used SAPs and the formation of hydrogels. The experimental part includes the presentation of data aimed at understanding the links between the mechanical performance of lime-based plasters and their microstructure.

## 2. Materials and Methods

### 2.1. Materials

A mixture of lime, cement, and sand in the weight ratio of 1:1:4 was used for the design of the reference lime-based plaster. Three grades of SAPs (Evonik, Essen, Germany) having different particle sizes and cross-linking densities were used for the modification of the reference mortar. The basic characteristics of used SAPs are summarized in Table 1.

**Figure 1:**
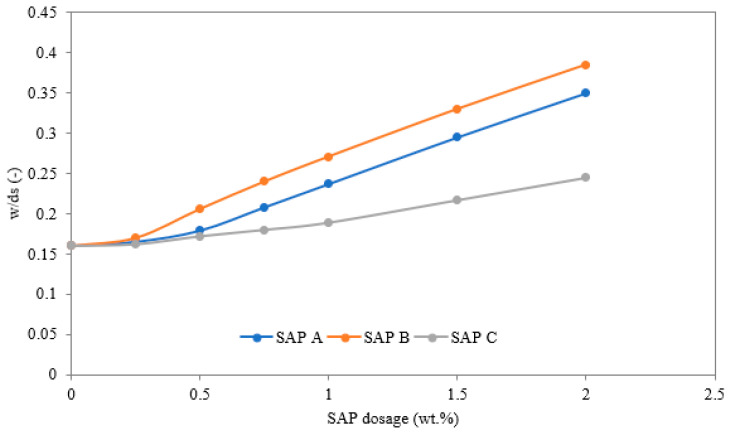
Relationship between SAP dosage and mass water/dry substance ratio for the spread diameter of 220 mm.

The reference lime-based plaster was modified by 0.5, 1, and 1.5 wt.% SAP dosages to reveal the material sensitivity to SAP amount. The mixtures were denoted as A (SAP A), B (SAP B), and C (SAP C) and further distinguished by numbers (for example A0.5) describing the applied SAP dosage. At the preparation of plaster mixtures, the water dosage was adjusted to keep a constant spread diameter of 220 mm, as determined by the flow table test. Figure 1 shows differences in water/dry substance (w/ds) ratio determined in preliminary experiments employed for the determination of the relationship between SAP dosage and extra water requirements. Here, the flow diameter of the fresh mixture was studied in consecutive steps, when the w/ds ratio was corrected in line with the increased content of selected SAP. For this experiment, a flow table experiment was used and a flow diameter of 220 mm was considered [26]. The obtained results pointed at an increased solubility of SAP C compared to the other two used SAP types, which affected the fresh mixture workability. The samples were cast into the molds, demolded after 2 days, and kept for 28 days in a highly humid environment (~95% RH). Detailed composition of studied materials is given in Table 2.

### 2.2. Experimental Methods

For the characterization of basic material properties, the measurement of bulk and matrix densities was carried out. First, samples were dried at 85 °C until a steady-state mass was reached. Afterward, five specimens were measured by a digital caliper to obtain the volume. The matrix density was determined with the help of a Pycnomatic ATC EVO helium pycnometer (Thermo Scientific, Waltham, MA, USA). Consequently, the total open porosity was calculated using the bulk and matrix densities. 

For material surface analysis, the dried samples were cut with a precise diamond saw and impregnated with a low viscosity resin (epoxy resin LH 289 and hardener H 289, Havel Composites CZ). A Struers Tegramin-20 automatic grinding and polishing machine (Struers Inc., Detroit, CL, USA) was used to grind the cured samples with a series of silicon carbide sandpapers of grit sizes 600, 1200, 2000, 2500, and 4000 for approximately 5 min each. Final hand polishing was performed using a diamond paste containing 0.25 μm particles. The prepared samples were dried in a vacuum desiccator for 2 days at 23 °C. After drying, the test samples were sputter-coated with gold/palladium using a Quorum SC7620 sputter coater (Quorum Technologies Ltd., Lewes, Great Britain).

The microstructure of the studied SAP-modified lime-based plasters was studied by scanning electron microscopy (SEM), using a Zeiss Merlin field emission gun scanning electron microscope (FEG-SEM, Zeiss, Jena, Germany) with a secondary electron detector operated at an acceleration voltage of 15 kV, probe current of 300–800 pA, and a working distance of 6–18 mm. The chemical composition of the material surface was determined by energy-dispersive X-ray spectroscopy (EDS).

The pore size distribution was characterized by mercury intrusion porosimetry (MIP) using Pascal 140 and Pascal 440 (Thermo Scientific, Waltham, MA, USA) devices. For the evaluation of results, a circular cross-section of capillaries was assumed, whereas the mercury contact angle was assumed to be 130°.

Compressive and flexural strengths were determined according to the ČSN EN 1015–11 standard after 28 days of curing in a highly humid environment. Flexural strength was measured by a three-point bending test on three specimens with the dimensions of 40 mm × 40 mm × 160 mm. Consequently, the compressive strength was measured on the left-over specimens after the flexural strength test.

## 3. Results and Discussion

**Figure 2:**
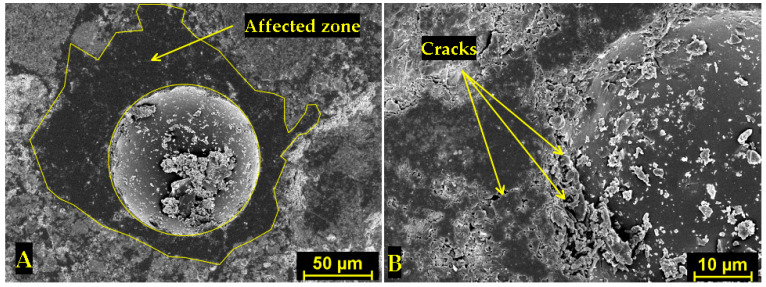
SEM image of the affected zone around the SAP core in hardened C1.5 mixture (**A**) and microcrack formation in ITZ (**B**).

**Figure 3:**
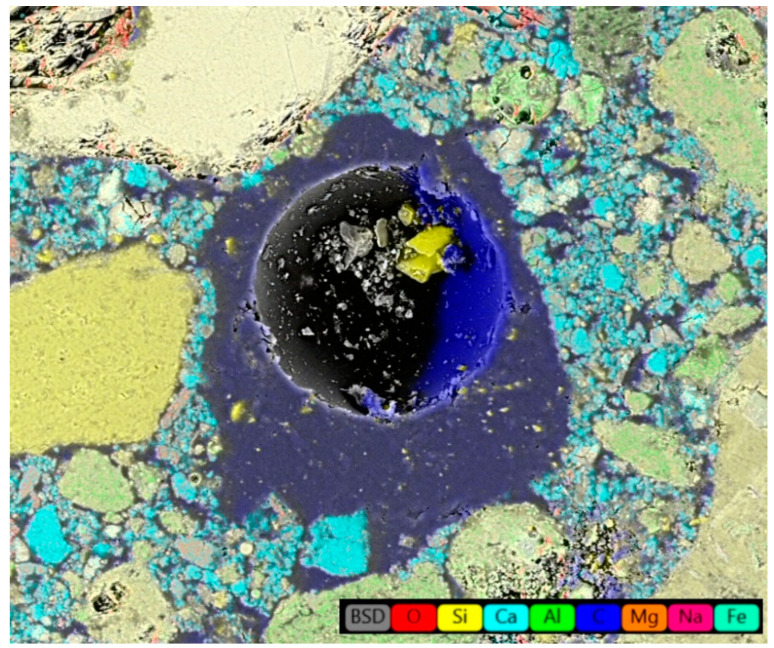
Element distribution map of hardened C1.5 mixture.

**Figure 4:**
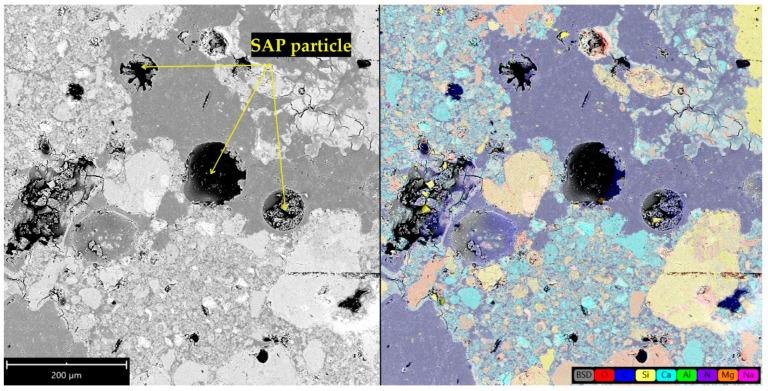
SEM image of the affected zone around the SAP core in hardened A1.5 mixture and element distribution map.

**Figure 5:**
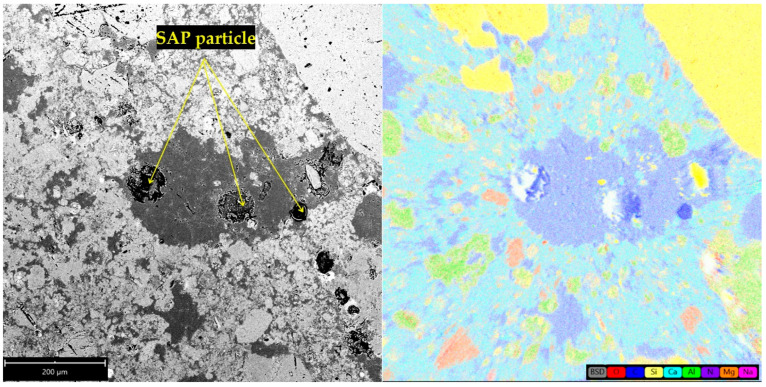
SEM image of the affected zone around the SAP core in hardened B1.5 mixture and element distribution map.

**Figure 6:**
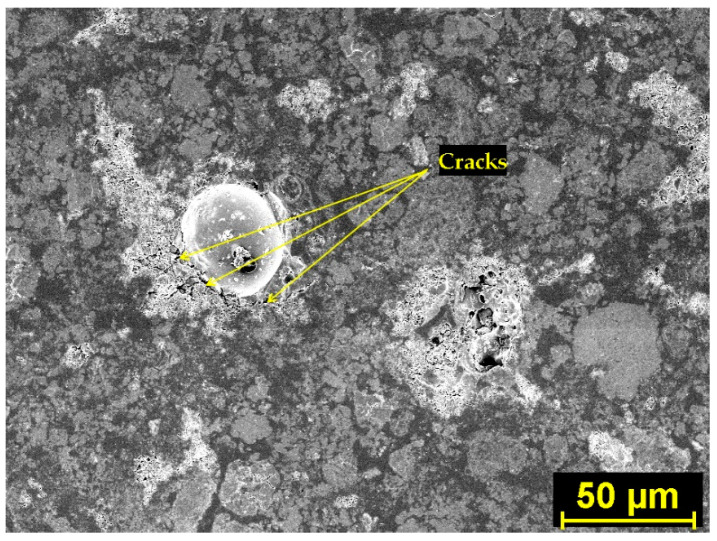
SEM image of microcracks in hardened B1.5 mixture around the SAP core.

The understanding of the effect of SAPs on microstructure development was found crucial for explaining the materials’ performance. In this regard, Figure 2 and Figure 3 show SAP cores in the C mixtures that created a layer of semisaturated hydrogel that could participate in internal curing and, at the same time, fill the pore space without significant particle clumping. Concurrently, some nucleation grains could be recognized in the swelled hydrogel, so the structure of hydrogel was not homogeneous. On the contrary, increased swelling characteristics of SAP A and SAP B induced particle clumping and formation of larger affected areas (see Figure 4 and Figure 5). The clumping occurrence indicates the nonproportional distribution of SAP particles and consequent weakening of the material’s structure. In other words, the increased swelling ratio poses a barrier to the successful and homogeneous incorporation of SAPs into the material matrix. The tendency for agglomeration was discussed previously for cement composites by Tenorio et al. [27] together with the susceptibility to changes in material microstructure and effects on mechanical strength. This factor is also relevant for the selection of optimal dosage with reduced adverse effects on functional performance. The heterogeneous structure and partial desiccation of hydrogel may also lead to microcrack formation on the interface of particular phases (Figure 6). This finding indicates a limited incorporation effectivity, which was observed also by Kang et al. [28] for cement composites.

The process of materials mixing causes partial or full saturation of absorption capacity of applied SAP particles, thus increasing their diameter. Therefore, the extra water dosage is necessary, as it was documented in Figure 1, to preserve the same workability of the fresh mixture. The different swelling capability in a water solution with other mix constituents is a major driving factor for the microstructure formation and hardened state properties. In particular, Ca^2+^ and Na^+^ have a significant effect on the SAP’s rate of solution absorption [29,30]. SAPs’ effectivity on internal curing or self-healing capability needs to be assessed cautiously since these factors may vary substantially if different SAP types are used.

The SEM images in this paper (Figure 2, Figure 3, Figure 4, Figure 5 and Figure 6) show that the microstructure of the analyzed lime-based plasters depended on the level of cross-linking density. The drying of SAPs was accelerated by higher cross-linking density as well as by small particles with a larger surface area compared to coarser particles. This was in accordance with the observations of Schrofl et al. [31] for cement matrices that lower cross-linking density values are indicative of decreased swelling and deswelling rates. The reduced occurrence of cracks can be linked with the cross-linking density of used SAPs considering also the findings of Zhu et al. [32].

However, the relationship between the inner structure and the applied SAP is two-fold. SAPs promote hydration and internal curing of nonhydrated particles; thus, their application has positive consequences [3]. On the other hand, SAPs create a so-called affected zone that may differ compared to the rest of the paste [33]. Nevertheless, the importance of the affected zone around SAP particles is not properly reflected in the available literature, for lime composites in particular. It should be noted that the quality of a description of the affected zone also depends on the experimental techniques used for materials characterization [34]. The affected zone may behave as an impermeable material, but it cannot be considered as the solid phase in full as it is not involved in the material strengthening [35].

Considering the principle of conventional methods used for composite materials analysis, the pore volume filled by SAP including the affected zone cannot be characterized sufficiently despite its particular importance and implication for functional properties. The formation of the affected zone is associated with the level of effective incorporation of a particular SAP into the material matrix and the influence of the desorption process of the SAP. The formation of the affected zone increases the degree of hydration and the hardness compared to the unaffected areas [34].

The water absorption governed by incorporated SAP particles could be also viewed as a factor that increases the compactness of paste in the surroundings thanks to the reduction in the local w/ds ratio. This effect may further result in crack formation (as can be seen in Figure 4 for the B1.5 mixture). Notwithstanding, this phenomenon depends on specific conditions around SAP particles and the water absorption rate. If SAP exhibits a lower absorption rate (this assumption is valid mostly for SAP C), the higher hydration degree can be considered, as can the occurrence of the limited cracks. Contrary to the findings by Liu et al. [36] who described the adverse effect of larger particle size on the mechanical strength of cement composites, in this paper the elucidation of this effect for lime-based plasters was found in the swelling rate rather than SAP particle size. Furthermore, the swelling rate depends strongly on the concentration of diluted ions (Ca^2+^, Na^+^, Al^3+^, etc.) and the covalent cross-linking density.

The carbon content in the specified area of lime-based plasters analyzed in this study corresponded to the chemical composition of all used SAPs based on polyacrylate/acrylamide/acrylic acid. The highlighted area in Figure 3 is in good agreement with the findings of Yang et al. [33] and Wang et al. [34], who described similar areas around SAP particles in cementitious materials. However, the area size and shape depend on the sample age [20].

**Figure 7:**
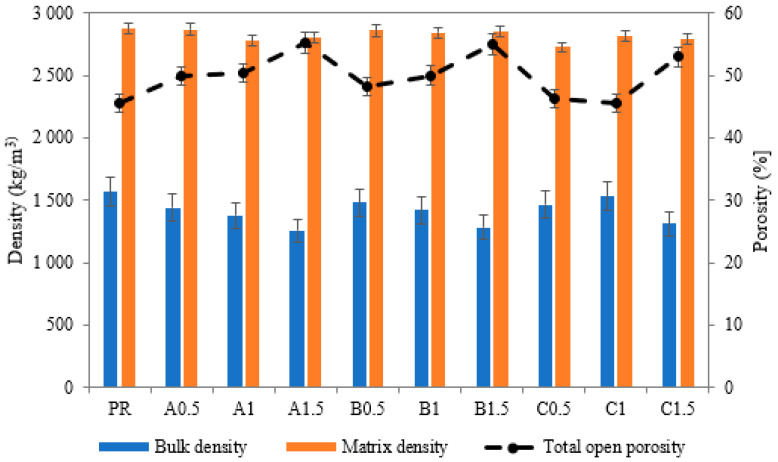
Basic material properties of lime-based plasters modified by SAPs.

The basic material properties are shown in Figure 7. The obtained results indicated that in general, the application of SAPs led to a decrease in bulk density and a corresponding increase in porosity. The matrix density remained on almost the same level, and only a small alteration induced by the lower density of applied SAPs could be observed. Taking into account the role of selected SAPs, the use of SAP A and SAP B caused very similar phenomena in terms of changes in the structure of the analyzed lime-based plasters. As the amount of SAP admixture increased, there was an increase in porosity and a reduction in bulk density compared to the reference sample. This effect contrasted with the application of SAP C, which resulted in an increase in porosity only in the case of 1.5 wt.% dosages. The explanation could be found in increased solubility and lowered viscosity of swelled SAP particles. Moreover, very fine SAP A particles together with a high cross-linking density increased the kinetics of liquid uptake to a greater extent compared to SAP C.

**Figure 8:**
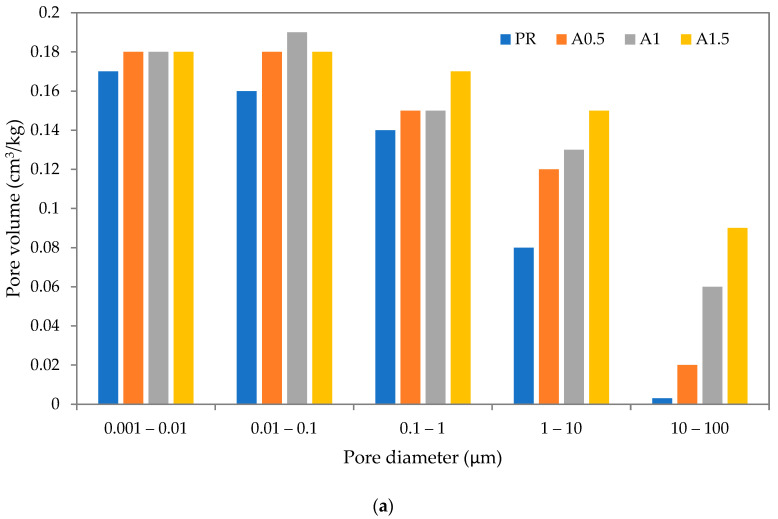

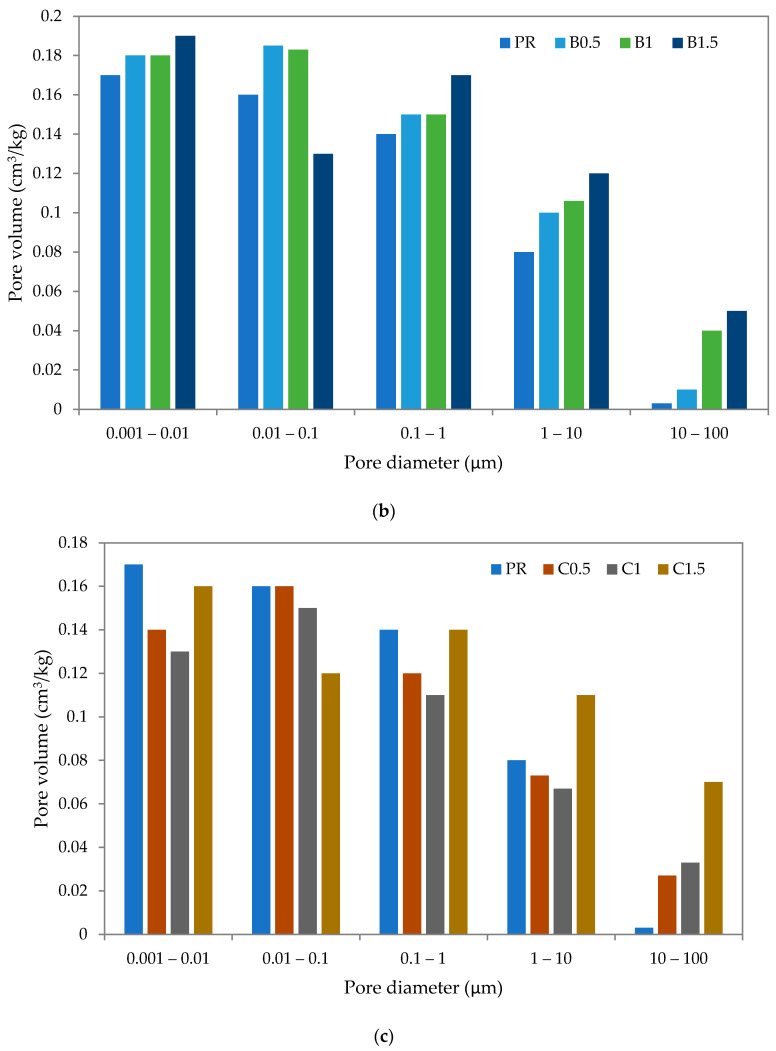
(**a**) Open pore size distribution of studied lime-based plasters with SAP A admixtures; (**b**) open pore size distribution of studied lime-based plasters with SAP B admixtures; (**c**) open pore size distribution of studied lime-based plasters with SAP C admixtures.

The data obtained by the MIP analysis showed differences in the pore size distribution caused by the incorporation of selected SAP types and dosages (see Figure 8a–c). As one can see, the utilization of SAPs in A and B mixtures resulted in an increased pore volume in the whole studied pore range (from 0.01 to 1000 μm). The changes were more pronounced for mixtures with higher content of SAPs. On the other hand, C mixtures exhibited a limited effect of the applied SAP. Even a minor reduction in pore volume in the range of 0.01–0.1 μm was noted in the case of 0.5 and 1 wt.% dosage. The explanation of this contradiction lies in the different swelling capabilities and solubilities in water of the particular SAPs. The most pronounced shift in pore volume was observed for A1.5 and B1.5 mixtures. It was induced by increased sorption capacity and higher susceptibility to particle agglomeration of SAPs A and B, which could affect SAP particle distribution and rheological properties of the mixes. The relationship between the pore diameter and degree of hydration described by Atahan et al. [37] indicates the significance of this parameter. Considering the effect of particle size, for the lime-based plasters in this paper, no relation was found between the particle size and pore size distribution, contrary to the results reported for cement pastes and composites by Lee et al. [4] and Ma et al. [38].

The amount of used cross-linker at the SAP production seems to be an important factor affecting the swelling capability of used SAPs and thus void formation in a material matrix, as was reported for cement composites by Jung et al. [39]. The swelling reduction of SAPs used in lime-based plasters analyzed in this paper was caused most likely by the physical restriction of the formed polymer chains due to the presence of physical cross-links and changes in the ion concentration. The cross-linking density had a substantial effect on the deswelling rate as well. The lime-based plasters modified by SAPs with lower particle diameter and higher cross-linking density exhibited a higher porosity.

The major factor for the proper understanding of the SAP role in the lime-based plasters studied in this paper was considering the changes in pore size distribution as a result of differences in material workability rather than leftover pores after SAP cores desorption. Farzanian and Ghahremaninezhad [40] pointed out that the desorption rate of SAPs in cementitious materials differed for hydrogels with contact with binder substrate compared to hydrogels without direct contact. Wehbe and Ghahremaninezhad [41] reported a distinct rim area around the affected zone connected by cracks with other voids. The explanation can be found in the beneficial effect of the Laplace pressure that pulls the hydrogel to a cementitious substrate and causes a decrease in the osmotic pressure near the interface, thus causing an increase in the desorption at the interface [42]. Additionally, the capillary sorption further boosts the hydrogel desorption and is followed by the hydration of nonhydrated binder particles and sealing of the formed space due to the volumetric expansion of hydration products. In other words, the desorption of swelled hydrogels does not contribute to the shift in the material porosity since the formed space is rather filled by hydration products, and SAP cores remain trapped in the center of the “affected zone”. In this regard, the application of SAPs with lower particle size and higher cross-linking density (SAP A) in this paper could be viewed as more prone to undesired modification of materials, while coarser particles and lower cross-linking density of SAP C did not cause such dramatic changes in the material microstructure.

It should be noted that the results obtained using the widely used MIP analysis should be subjected to critical consideration, taking into account the desorption characteristics of SAP particles. Once SAPs are used as an admixture, they consume a significant amount of water and swell considerably [43]. SAPs create voids filled by hydrogel in the microstructure of cementitious materials and contribute to internal curing, mitigation of autogenous shrinkage, and sealing of formed cracks. Serious doubt about using high intrusion pressures applied by MIP for the study of very small pores was therefore expressed by Snoeck et al. [35]. On this account, the utilization of experimental alternatives, such as dynamic vapor sorption and cryoporometry, was found as more effective for microstructure studies. Notwithstanding, the conclusions in most research papers did not include the interaction of swelled SAP particles with the surrounding matrix and further formation of the affected zone [44,45,46]. On top of that, swelled SAP particles themselves cannot be penetrated by the most of measuring media, thus remaining hidden for common techniques. Taking into account the swelled volume of SAPs, such interpretation may distort the presented data, especially in a relation to mechanical strength and specifically for high absorption SAPs with susceptibility to agglomeration of SAP particles. This effect is more noticeable in the plaster design where it is manifested by higher water dosage.

Taking into account the aforementioned results, the presence of SAPs remains hidden when conventional methods such as MIP or DVS are employed. Therefore, such methods are not able to identify the volume of secondary pores created by the SAP admixture. However, the contribution of SAP particles to the worsening of mechanical strength can be compared with the effect of closed pores in a material microstructure. Although the relatively low volume of applied SAP admixtures could be viewed as unimportant, taking into account the extra water dosage consumed by swelling increases considerably the volume of the formed hydrogel. Therefore, the role of SAPs in understanding material performance and its effects on mechanical strength should be reconsidered.

**Figure 9:**
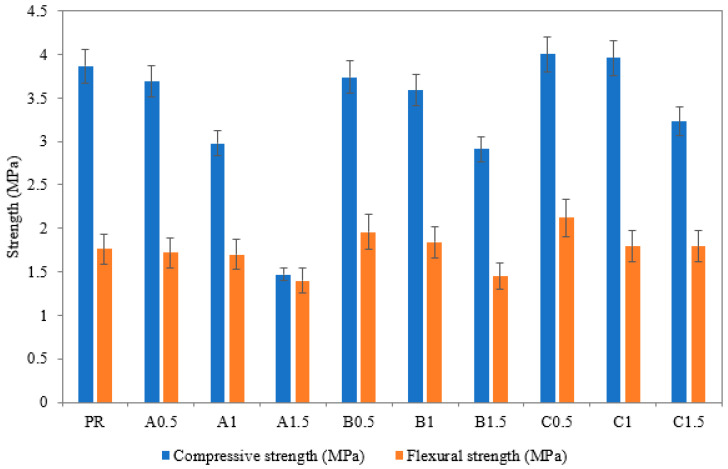
Compressive and flexural strengths of studied lime-based plasters.

Compressive and flexural strengths of the analyzed lime-based plasters are presented in Figure 9. Here, very similar effects were noted for SAP A and SAB B mixtures. Specifically, the application of both SAPs resulted in a gradual reduction in monitored strength parameters with the increasing SAP dosage. Notwithstanding, SAP B incorporation led to a lower strength decrease compared to SAP A. This result could be attributed to different sorption capabilities of SAPs that had an impact on the fresh mixture rheology and the amount of created voids. On the other hand, the application of SAP C was found positive for mechanical strength up to 1 wt.% SAP dosage. The beneficial effect of SAP C incorporation is due to improved internal curing and prolongation of the hydration period, which was reported in research papers dealing with self-healing concrete design [47,48]. All lime-based mixtures showed a significant decrease in mechanical strength for 1.5 wt.% dosages. Therefore, the 1% SAP dosage can be viewed as a threshold value for the preservation of mechanical properties. Viewing the rapid swelling as the principal barrier for strength preservation, future research efforts aimed at SAP coating to decrease initial rapid swelling may provide more satisfactory results in terms of mechanical strength. 

**Figure 10:**
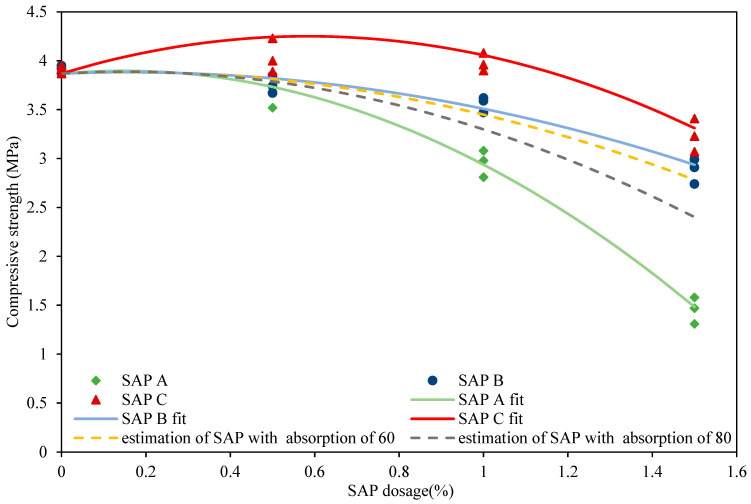
Analytical expressions of the dependence of compressive strength of studied lime-based plasters on SAP dosage and SAP water absorption capacity.

The dependence of compressive strength on the four SAP dosages *x* = 0, 0.5, 1, and 1.5 wt.% can be well approximated by a quadratic curve *ax*^2^ + *bx* + *c* for each of the three mixtures A, B, and C with the constants *a* and *b* differing for each mixture and *c* being the same. Thus, both *a*(*y*) and *b*(*y*) change with the water absorption capacity *y* of the mixtures (their values are given in Table 1). If we wish to predict the compressive strength for a mixture of a different absorption capacity, *y*_0_, we may obtain the corresponding quadratic curve *a*_0_*x*^2^ + *b*_0_*x* + *c* by determining the constants *a*_0_ and *b*_0_ from the piecewise linear approximations of the obtained dependences *a*(*y*) and *b*(*y*) calculated at *y* = *y*_0_. The analytical expressions of the dependence of compressive strength of studied lime-based plasters on SAP dosage and SAP water absorption capacity are summarized in Figure 10.

For cement composites, a shift in material porosity, the macroporosity in particular, as a result of desorption of swelled SAP particles is considered as the apparent reason for the strength decrease [49]. On the other hand, the generally accepted conclusions obtained by intensive investigation of the effect of SAPs on material microstructure identified the densification of the cementitious matrix due to the continuous hydration caused by SAP-released water [50,51]. The effect of densification was more evident in areas surrounding SAP, similar to the lowered volume of capillary pores. The formed microvoids in areas surrounding SAP are often sealed by swelled hydrogel which prevents their proper identification by intrusion techniques. In this regard, SAP studies aimed at the utilization of SAPs as sealing agents in cement composites showed a rapid capability to close cracks formed by their swelling [52]. A similar conclusion was also formulated by Kang et al. [28], who reported that the majority of pores were not connected to each other and separated independently. The particular importance of the discussed phenomenon lies in the formation of the increased number of closed pores that inflict a reduction in mechanical strength. Formed closed pores are not completely empty but fully or partially filled by swelled hydrogel particles that form a very dense matrix on their edges. However, such pores, as well as free voids, weaken the mechanical parameters of cement composites to a great extent [53]. The mechanical strength drop cannot thus be clearly attributed only to the increased amount of interconnected pores formed, which is usually caused by changes in the fresh mixtures’ workability. The amount and distribution of pores are considered as limiting factors for the strength of composite materials, and the presence of larger pores poses a negative phenomenon that may limit the application of SAP-modified materials. According to the available literature, the surrounding zone around SAP particles and their formation is essential for the hardened state properties of cement composites, the strength in particular [54]. 

The investigations of microstructure and mechanical performance of lime-based plasters in this paper showed that the utilization of tailor-made SAPs can be a very effective tool. The choice of appropriate particle size diameter and cross-linking density of SAPs can provide satisfactory results in terms of extended functional performance (improved internal curing, moisture moderation, crack self-healing, etc.) achieved with only limited worsening of mechanical parameters. 

## 4. Conclusions

The effect of superabsorbent polymers (SAPs) on the microstructure formation and mechanical performance of lime-based plasters was studied in the paper. The chemical composition of the SAPs, together with the particle size and cross-linking density, was taken into account. The following conclusions can be drawn from the results obtained in the study:

The utilization of SAPs with higher cross-linking density modified the microstructure of designed lime-based plasters to a greater extent compared to coarser SAPs with reduced cross-linking density.

Coarser SAPs with low cross-linking density in the dosage of 0.5 and 1 wt.% were found as the most suitable solution, leading even to a slight improvement of mechanical parameters. The incorporation of SAPs with higher cross-linking density reduced the mechanical performance even for small dosages of 0.5 wt.%.

The increased swelling capability of used SAP tends to result in undesired agglomeration of SAP particles that may result in nonuniform SAP distribution and consequently weaken the microstructure of the plaster.

Different characteristics of the affected zone around SAP particles provided important information for proper understanding of the structure of SAP-modified lime-based plasters in the sense of swelled hydrogel presence and its incorporation into the matrix microstructure.

The effect of secondary porosity formed by swelled or semiswelled hydrogels can be considered as an underestimated parameter as yet considering the high swelling capability of SAPs. The voids filled by hydrogel are difficult to detect by conventional experimental techniques, and the tendency of SAPs to fill closely related cracks may thus not be taken into account properly. However, the neglect of this phenomenon may result in incorrect material parameter interpretation. The role of porosity in building materials is essential in various aspects, including the mechanical performance and moisture/heat transport processes. This effect will be more pronounced for low-porosity composites, where SAPs are extensively investigated as shrinkage mitigation agents.

In this regard, follow-up research should be aimed at the identification of an efficient experimental technique applicable for the determination of air- and SAP-filled voids. Consequently, a detailed investigation of the SAP-affected zone may provide advanced knowledge of effective dosage considering the shrinkage mitigation and internal curing efficiency to reach the equilibrium between the material performance and costs.

## Figures and Tables

**Table 1:** Characteristics of used superabsorbent polymers.

	Particle Size (µm)	Density (kg/m^3^)	Water Absorption Capacity (g/g)	Cross-Linking (wt.%)	Base
SAP A	285	600	128	1.2	acrylamide/acrylic acid potassium salt
SAP B	32	690	52	1	sodium polyacrylate
SAP C	84	605	45	0.3	acrylamide/acrylic acid + sodium salt

**Table 2:** Composition of studied plasters.

Mixture	Dry Plaster Mixture (kg/m^3^)	w/ds	SAP
		(-)	
PR	1351	0.16	-
A0.5	1255	0.177	0.5%
A1	1161	0.225	1.0%
A1.5	1060	0.276	1.5%
B0.5	1230	0.205	0.5%
B1	1117	0.271	1.0%
B1.5	1004	0.326	1.5%
C0.5	1274	0.167	0.5%
C1	1277	0.175	1.0%
C1.5	1185	0.189	1.5%

## Data Availability

Not applicable.

## References

[1] Fernandez C.A., Correa M., Nguyen M.T., Rod K.A., Dai G.L., Cosimbescu L., Rousseau R., Glezakou V.A. (2021). Progress and challenges in self-healing cementitious materials. J. Mater. Sci..

[2] Makul N. (2020). Advanced smart concrete—A review of current progress, benefits and challenges. J. Clean. Prod..

[3] He Z.M., Shen A.Q., Guo Y.C., Lyu Z.H., Li D.S., Qin X., Zhao M., Wang Z.L. (2019). Cement-based materials modified with superabsorbent polymers: A review. Constr. Build. Mater..

[4] Lee H.X.D., Wong H.S., Buenfeld N.R. (2016). Self-sealing of cracks in concrete using superabsorbent polymers. Cem. Concr. Res..

[5] Hong G., Song C., Choi S. (2020). Autogenous Healing of Early-Age Cracks in Cementitious Materials by Superabsorbent Polymers. Materials.

[6] Fort J., Koci J., Pokorny J., Cerny R. (2020). Influence of Superabsorbent Polymers on Moisture Control in Building Interiors. Energies.

[7] Fort J., Sal J., Koci J., Cerny R. (2020). Energy Efficiency of Novel Interior Surface Layer with Improved Thermal Characteristics and Its Effect on Hygrothermal Performance of Contemporary Building Envelopes. Energies.

[8] Vieira J., Senff L., Goncalves H., Silva L., Ferreira V.M., Labrincha J.A. (2014). Functionalization of mortars for controlling the indoor ambient of buildings. Energy Build..

[9] Senff L., Ascensao G., Hotza D., Ferreira V.M., Labrincha J.A. (2016). Assessment of the single and combined effect of superabsorbent particles and porogenic agents in nanotitania-containing mortars. Energy Build..

[10] Ma S.W., Huang C.H., Baah P., Nantung T., Lu N. (2021). The influence of water-to-cement ratio and superabsorbent polymers (SAPs) on solid-like behaviors of fresh cement pastes. Constr. Build. Mater..

[11] Kanellopoulou I.A., Kartsonakis I.A., Charitidis C.A. (2021). The Effect of Superabsorbent Polymers on the Microstructure and Self-Healing Properties of Cementitious-Based Composite Materials. Appl. Sci..

[12] Luo M., Bai J.Q., Jing K., Ding Z.Q., Yang D.Y., Qian C.X. (2021). Self-healing of early-age cracks in cement mortars with artificial functional aggregates. Constr. Build. Mater..

[13] Huang H.L., Ye G. (2015). Self-healing of cracks in cement paste affected by additional Ca^2+^ ions in the healing agent. J. Intell. Mater. Syst. Struct..

[14] Sidiq A., Gravina R., Setunge S., Giustozzi F. (2020). The effectiveness of Super Absorbent polymers and superplasticizer in self-healing of cementitious materials. Constr. Build. Mater..

[15] Zhao S.Y., Jensen O.M., Hasholt M.T. (2020). Measuring absorption of superabsorbent polymers in cementitious environments. Mater. Struct..

[16] Lefever G., Aggelis D.G., De Belie N., Raes M., Hauffman T., Van Hemelrijck D., Snoeck D. (2020). The Influence of Superabsorbent Polymers and Nanosilica on the Hydration Process and Microstructure of Cementitious Mixtures. Materials.

[17] Liu J.H., Farzadnia N., Khayat K.H., Shi C.J. (2021). Effects of SAP characteristics on internal curing of UHPC matrix. Constr. Build. Mater..

[18] Reis P.F.O., Evangelista F.E., Silva E.F. (2020). Profile of internal relative humidity and depth of drying in cementitious materials containing superabsorbent polymer and nano-silica particles. Constr. Build. Mater..

[19] Yang L., Shi C.J., Wu Z.M. (2019). Mitigation techniques for autogenous shrinkage of ultra-high-performance concrete—A review. Compos. Part B-Eng..

[20] Yang J.B., Sun Z.P., Zhao Y.H., Ji Y.L., Li B.Y. (2020). The Water Absorption-release of Superabsorbent Polymers in Fresh Cement Paste: An NMR Study. J. Adv. Concr. Technol..

[21] Mechtcherine V., Snoeck D., Schroefl C., De Belie N., Klemm A.J., Ichimiya K., Moon J., Wyrzykowski M., Lura P., Toropovs N. (2018). Testing superabsorbent polymer (SAP) sorption properties prior to implementation in concrete: Results of a RILEM Round-Robin Test. Mater. Struct..

[22] Senff L., Modolo R.C.E., Ascensao G., Hotza D., Ferreira V.M., Labrincha J.A. (2015). Development of mortars containing superabsorbent polymer. Constr. Build. Mater..

[23] Goncalves H., Goncalves B., Silva L., Raupp-Pereira F., Senff L., Labrincha J.A. (2014). Development of porogene-containing mortars for levelling the indoor ambient moisture. Ceram. Int..

[24] Goncalves H., Goncalves B., Silva L., Vieira N., Raupp-Pereira F., Senff L., Labrincha J.A. (2014). The influence of porogene additives on the properties of mortars used to control the ambient moisture. Energy Build..

[25] Isikdag B., Topcu I.B. (2013). The effect of ground granulated blast-furnace slag on properties of Horasan mortar. Constr. Build. Mater..

[26] Fort J., Madera J., Hotek P., Mildner M., Cerny R. Optimization of Concrete Mixture Composition with Superabsorbent Polymer Admixture. Proceedings of the International Conference on Numerical Analysis and Applied Mathematics (ICNAAM).

[27] Tenorio J.R., Snoeck D., De Belie N. (2020). Mixing protocols for plant-scale production of concrete with superabsorbent polymers. Struct. Concr..

[28] Kang S.H., Hong S.G., Moon J. (2018). The effect of superabsorbent polymer on various scale of pore structure in ultra-high performance concrete. Constr. Build. Mater..

[29] Choi H., Inoue M., Kim D., Sengoku R. (2019). Effect of Addition of Ca^2+^ and CO_3_^2−^ Ions with Temperature Control on Self-Healing of Hardened Cement Paste. Materials.

[30] Hu M.M., Guo J.T., Du J.B., Liu Z.X., Li P.P., Ren X.K., Feng Y.K. (2019). Development of Ca^2+^-based, ion-responsive superabsorbent hydrogel for cement applications: Self-healing and compressive strength. J. Colloid Interface Sci..

[31] Schrofl C., Mechtcherine V., Gorges M. (2012). Relation between the molecular structure and the efficiency of superabsorbent polymers (SAP) as concrete admixture to mitigate autogenous shrinkage. Cem. Concr. Res..

[32] Zhu Q., Barney C.W., Erk K.A. (2015). Effect of ionic crosslinking on the swelling and mechanical response of model superabsorbent polymer hydrogels for internally cured concrete. Mater. Struct..

[33] Yang J., Wang F.Z., He X.Y., Su Y. (2019). Pore structure of affected zone around saturated and large superabsorbent polymers in cement paste. Cem. Concr. Compos..

[34] Wang F.Z., Yang J., Hu S.G., Li X.P., Cheng H. (2016). Influence of superabsorbent polymers on the surrounding cement paste. Cem. Concr. Res..

[35] Snoeck D., Pel L., De Belie N. (2019). Comparison of different techniques to study the nanostructure and the microstructure of cementitious materials with and without superabsorbent polymers. Constr. Build. Mater..

[36] Liu H.Z., Zhang Q., Gu C.S., Su H.Z., Li V. (2017). Influence of microcrack self-healing behavior on the permeability of Engineered Cementitious Composites. Cem. Concr. Compos..

[37] Atahan H.N., Oktar O.N., Tasdemir M.A. (2009). Effects of water-cement ratio and curing time on the critical pore width of hardened cement paste. Constr. Build. Mater..

[38] Ma X.W., Liu J.H., Wu Z.M., Shi C.J. (2017). Effects of SAP on the properties and pore structure of high performance cement-based materials. Constr. Build. Mater..

[39] Jung A., Endres M.B., Weichold O. (2020). Influence of Environmental Factors on the Swelling Capacities of Superabsorbent Polymers Used in Concrete. Polymers.

[40] Farzanian K., Ghahremaninezhad A. (2018). Desorption of superabsorbent hydrogels with varied chemical compositions in cementitious materials. Mater. Struct..

[41] Wehbe Y., Ghahremaninezhad A. (2017). Combined effect of shrinkage reducing admixtures (SRA) and superabsorbent polymers (SAP) on the autogenous shrinkage, hydration and properties of cementitious materials. Constr. Build. Mater..

[42] Krafcik M.J., Erk K.A. (2016). Characterization of superabsorbent poly(sodium-acrylate acrylamide) hydrogels and influence of chemical structure on internally cured mortar. Mater. Struct..

[43] Patra S.K., Swain S.K. (2011). Swelling Study of Superabsorbent PAA-co-PAM/Clay Nanohydrogel. J. Appl. Polym. Sci..

[44] Snoeck D., Dewanckele J., Cnudde V., De Belie N. (2016). X-ray computed microtomography to study autogenous healing of cementitious materials promoted by superabsorbent polymers. Cem. Concr. Compos..

[45] Zhong P.H., Wyrzykowski M., Toropovs N., Li L., Liu J.P., Lura P. (2019). Internal curing with superabsorbent polymers of different chemical structures. Cem. Concr. Res..

[46] Tan Y.W., Chen H.X., Wang Z.D., Xue C., He R. (2019). Performances of Cement Mortar Incorporating Superabsorbent Polymer (SAP) Using Different Dosing Methods. Materials.

[47] Danish A., Mosaberpanah M.A., Salim M.U. (2021). Robust evaluation of superabsorbent polymers as an internal curing agent in cementitious composites. J. Mater. Sci..

[48] Paul A., Murgadas S., Delpiano J., Moreno-Casas P.A., Walczak M., Lopez M. (2021). The role of moisture transport mechanisms on the performance of lightweight aggregates in internal curing. Constr. Build. Mater..

[49] Dang J.T., Zhao J., Du Z.H. (2017). Effect of Superabsorbent Polymer on the Properties of Concrete. Polymers.

[50] Shen D.J., Wang X.D., Cheng D.B., Zhang J.Y., Jiang G.Q. (2016). Effect of internal curing with super absorbent polymers on autogenous shrinkage of concrete at early age. Constr. Build. Mater..

[51] Guo S.C., Forooshani P.K., Dai Q.L., Lee B.P., Si R.Z., Wang J.Q. (2020). Design of pH-responsive SAP polymer for pore solution chemistry regulation and crack sealing in cementitious materials. Compos. Part B-Eng..

[52] Gwon S., Ahn E., Shin M. (2020). Water permeability and rapid self-healing of sustainable sulfur composites using superabsorbent polymer and binary cement. Constr. Build. Mater..

[53] Olawuyi B.J., Babafemi A.J., Boshoff W.P. (2021). Early-age and long-term strength development of high-performance concrete with SAP. Constr. Build. Mater..

[54] Tan Y.W., Lu X.S., He R., Chen H.X., Wang Z.J. (2021). Influence of superabsorbent polymers (SAPs) type and particle size on the performance of surrounding cement-based materials. Constr. Build. Mater..

